# A Novel Low-Activation VCrFeTa*_x_*W*_x_* (*x* = 0.1, 0.2, 0.3, 0.4, and 1) High-Entropy Alloys with Excellent Heat-Softening Resistance

**DOI:** 10.3390/e20120951

**Published:** 2018-12-11

**Authors:** Weiran Zhang, Peter K. Liaw, Yong Zhang

**Affiliations:** 1 State Key Laboratory for Advanced Metals and Materials, University of Science and Technology Beijing, Beijing 100083, China; 2 Department of Materials Science and Engineering, The University of Tennessee, Knoxville, TN 37996, USA; 3 Beijing Advanced Innovation Center for Materials Genome Engineering, University of Science and Technology Beijing, Beijing 100083, China

**Keywords:** low-activation high-entropy alloys (HEAs), high-temperature structural alloys, microstructures, compressive properties, heat-softening resistance

## Abstract

The microstructure, Vickers hardness, and compressive properties of novel low-activation VCrFeTa*_x_*W*_x_* (*x* = 0.1, 0.2, 0.3, 0.4, and 1) high-entropy alloys (HEAs) were studied. The alloys were fabricated by vacuum-arc melting and the characteristics of these alloys were explored. The microstructures of all the alloys exhibited a typical morphology of dendritic and eutectic structures. The VCrFeTa_0.1_W_0.1_ and VCrFeTa_0.2_W_0.2_ alloys are essentially single phase, consisting of a disordered body-centered-cubic (BCC) phase, whereas the VCrFeTa_0.2_W_0.2_ alloy contains fine, nanoscale precipitates distributed in the BCC matrix. The lattice parameters and compositions of the identified phases were investigated. The alloys have Vickers hardness values ranging from 546 HV_0.2_ to 1135 HV_0.2_ with the x ranging from 0.1 to 1, respectively. The VCrFeTa_0.1_W_0.1_ and VCrFeTa_0.2_W_0.2_ alloys exhibit compressive yield strengths of 1341 MPa and 1742 MPa, with compressive plastic strains of 42.2% and 35.7%, respectively. VCrFeTa_0.1_W_0.1_ and VCrFeTa_0.2_W_0.2_ alloys have excellent hardness after annealing for 25 h at 600–1000 °C, and presented compressive yield strength exceeding 1000 MPa with excellent heat-softening resistance at 600–800 °C. By applying the HEA criteria, Ta and W additions into the VCrFeTaW are proposed as a family of candidate materials for fusion reactors and high-temperature structural applications.

## 1. Introduction

With the rapid development of human civilization, the demand for energy is increasing and the fossil fuel sources are running out. As nuclear energy produces more energy with less pollution, in the long run, nuclear energy will be the next major energy source after fossil fuels, such as coal and oil, to meets human needs [1,2,3]. However, with fast-growing nuclear power technology, people have higher requirements for the reliability and safety of the nuclear-power [4,5]. The structural materials for new commercial fusion nuclear reactors operate in a harsh environment that is high temperature and chemically reactive, and experiences time-varying stress and intense neutron radiation [4,6], while required to be environmentally-friendly (reduced activation properties). This has motivated worldwide research and development (R&D) on advanced nuclear power systems. Therefore, the exploitation of novel and advanced materials that meet the requirements of these severe conditions will be a key issue for the development of new commercial reactors in the future [4].

Reduced activation or low-activation materials means that the main source of radioactivity after neutron irradiation is short- or medium-lived radioactive elements [7]. The challenge is managing the radioactive waste after shutting down the reactor, and fusion will lose its advantage of being a cleaner energy. Hence, it is important to choose specific materials for the reliable operation of these reactors [8]. The reduced activation ferritic/martensitic (RAFM) steels [9,10,11], such as Eurofer 97, China low activation martensitic (CLAM), and F82H, are considered to be the original candidate blanket structural materials and/or first wall for future fusion-power devices due to their excellent thermophysical properties, high thermomechanical capabilities, low-activation property, and resistance to neutron irradiation. RAFM steels have been developed using modified (8–12)CrMoVNb type ferritic martensitic steels by replacing Nb, Mo, and Ni with W, Mn, and Ta to achieve the low activation properties [1,7]. However, the operating temperature limit of RAFM steels is currently about 550 °C, which limits the overall thermodynamic efficiency of the power plant [12]. In order to widen the operating temperature window for fusion reactors, several alternative advanced materials options are being pursued. These alternatives include oxide-dispersion-strengthened (ODS) ferritic steels [5], vanadium alloys, and silicon carbide fiber-reinforced silicon carbide matrix composites [13,14]. Existing materials struggle to meet the requirements of fusion reactors operating in extreme environments, such as higher temperatures and stronger neutron irradiation [13,15,16,17]. Therefore, the first task for the development of fusion energy is to develop high-performance materials. According to the requirements of fusion reactors for short- or medium-lived radioactive materials and existing alloys, such as RAFM steels, ODS steels, and vanadium alloys, we summarize the low activation elements and high activation elements in Table 1.

High-entropy alloys (HEAs) are new materials developed in the field of metals in the past decade [18,19,20]. The term HEAs signifies unconventional alloy systems composed of at least four principal elements, and the atomic percent of each composed element is between 5 at. % (atomic percent) and 35 at. %, which benefits the formation of single-phase solid-solution on the simple underlying face-centered-cubic (FCC), body-centered-cubic (BCC), and hexagonal-close-packing (HCP) structures compared with intermetallics [19,21,22]. HEAs are strongly contrasted with conventional alloys, which are usually based on one or two major elements, and the addition of trace amounts of alloying elements mostly leads to the formation of new phases [4,23]. HEAs can have high hardness [24,25,26,27], great creep resistance [28,29], good irradiation resistance [4,6,16,30], good structural stability [28,31,32,33,34], and excellent high-temperature strength [28,31,35]. These advantages make HEAs specifically suitable for high-temperature [13] and irradiation applications [14,30]. Zhang et al. [15] reported that Al*_x_*CoCrFeNi (*x* = 0.1, 0.75, and 1.5) shows great phase stability and swelling resistance under heavy ion irradiation at room temperature to high displacement per atom (dpa). They thought that this trend is due to the severe lattice distortion and sluggish diffusion, which are unique to HEAs. MoNbHfZrTi [36] shows a high compressive yield strength of 1719 MPa and 1575 MPa in as-cast and as-homogenized states at room temperature, respectively. This alloy has high compressive yield strength at elevated temperatures (825 MPa at 800 °C and 187 MPa at 1200 °C) and shows a drop in flow stress after yielding.

The study of HEAs has expanded from the central region to the surroundings of the phase diagram, which means that the research is changing from examining equiatomic single-phase solid-solution alloys to non-equimolar multi-phase solid-solution alloys [9,22,37,38]. According to the concept of HEAs, the reduced activation elements of Fe, Cr, V, W, and Ta were chosen to form several non-equimolar and an equiatomic TaWFeCrV HEAs. However, W is a candidate element for plasma-oriented components in commercial energy-fusion reactors, as it can increase strength and reduce the brittle transition temperature [3,39,40,41]. Cr reinforces the corrosion resistance of the alloys [5,42,43]. V and Ta can improve creep properties, reduce grain size, and enhance the toughness and strength of the alloys [44,45]. Fe has excellent ductility [46] and is inexpensive. In this study, the V–Cr–Fe–Ta–W alloys were prepared by vacuum-arc melting. Their alloying behaviors, microstructures, and mechanical properties were investigated in detail.

## 2. Experimental Procedures

Alloy ingots with the nominal composition of VCrFeTa*_x_*W*_x_* (*x*: molar ratio; *x* = 0.1, 0.2, 0.3, 0.4, and 1 denoted by T0.1, T0.2, T0.3, T0.4, and T1, respectively) were prepared by vacuum arc melting with high purity elements (the purity of each elements was better than 99.9 wt. %) in a Ti-gettered high-purity argon atmosphere. The nominal chemical compositions of the obtained five alloys are listed in Table 2. These ingots were re-melted at least 5 times in order to achieve compositional homogeneity, and each sample weight was around 50 g. The produced alloys were annealed at 800 °C for 25 h in a high-purity argon furnace and cooled down in water.

The analyzed samples were cut from the middle part of the as-cast alloys to attain flat surfaces for the microstructure study. The exposed surfaces were subsequently ground and polished with the standard polishing process. The crystal structures of as-cast and annealed samples were identified by X-ray diffraction (XRD) (Bruker D8, Karlsruhe, Germany.) with Cu Kα radiation generated at 40 kV and 40 mA and with the scanning angles (2θ) ranging from 30° to 100° at a step of 0.0102° and 10 s dwell time per step. A scanning electron microscope (SEM) (ZEISS SUPRA 55, Jena, Germany.) was used for microstructural analyses, and experimental compositions were analyzed by the energy-dispersive spectroscopy (EDS) (ZEISS SUPRA 55, Jena, Germany.).

Mechanical properties were studied in terms of compression and micro-hardness in air at room temperature. The cylindrical test specimens with a diameter of 3 mm and height of 6 mm were cut from the middle of the ingots for compressive tests, which were performed on a computer-controlled electronic universal testing machine. The high-temperature compressive performances were studied on a Gleeble machine. The heating rate was set to 20 °C/s, and the holding time was five minutes, then air cooled. The initial strain rate for all compressive tests was 10^−3^ s^−1^. The Vickers micro-hardness tests were carried out with a load of 200 g and a 15 s dwelling time with at least 12 tested points for each test specimen. SEM was used to observe the fracture surfaces of the samples after compressive tests.

## 3. Results

### 3.1. Structural Characterization

**Figure 1:**
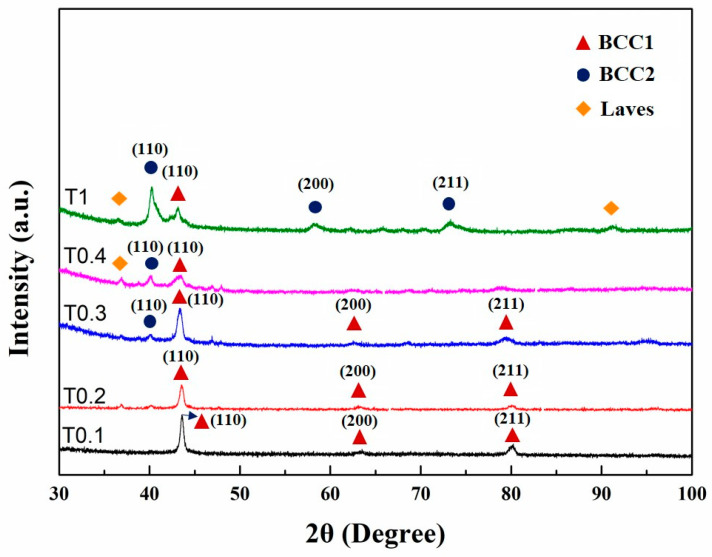
X-ray diffraction (XRD) patterns of the as-cast VCrFeTa*_x_*W*_x_* (*x* = 0.1, 0.2, 0.3, 0.4, and 1) alloys.

The XRD patterns of as-cast alloys are shown in Figure 1. Only one BCC1 crystal structure (the Fe-, Cr-, and V-rich phase) had lattice parameters of 0.2935 nm and 0.2937 nm, which were identified in T0.1 and T0.2 alloys in the as-cast state according to Bragg’s law, respectively. With the increase in Ta and W content, the structures became rather complex. Both BCC1 and BCC2 (W-rich phase) solid-solution structures, together with Laves (Fe_2_Ta-type) phases, were found in these alloys. When the molar ratio of Ta and W increased to 0.3, BCC2 appeared with a lattice parameter of 0.3174 nm. The lattice parameter of the BCC1 phase was 0.2947 nm with a peak width much wider than those of the T0.1 and T0.2 alloys. However, there was a weaker peak of the Laves phase around 47°. The phase composition of T0.4 is similar to T0.3, with the only difference in the strength. The peak (110) for BCC1 is broader than that of the T0.3, and the peak intensity is weaker. The peak of BCC2 phase with the lattice parameter of 0.3178 nm is stronger, both in the peak width and intensity compared with T0.3. Despite this trend, T0.4 is mainly composed of BCC1 with a lattice parameter of 0.2963 nm. When the alloy reaches an equimolar ratio, VCrFeTaW, which is denoted T1, is composed of two BCC phases with lattice parameters of 0.2962 nm for BCC1 and 0.3166 nm for BCC2.

Comparing the X-ray diffraction parameters of each sample, the lattice parameter of BCC1 first rises and then falls, as shown in Figure 1. When the contents of Ta and W reached 0.4 molar, the lattice constants of BCC1 and BCC2 were the largest. The lattice-constant changing trend of BCC2 is similar to that of BCC1. The difference is that the decreasing degree of BCC2 is greater than BCC1. It can be seen from the XRD pattern that the Laves phase has obvious diffraction peaks when *x* reaches 0.4. The intensity of the diffraction peak of BCC1 decreased significantly but still dominated. The phase composition and lattice constant of each alloy are listed in Table 3. We explain this phenomenon in two ways. Considering the atomic radius, the atomic radii of Ta and W are 1.48 Å and 1.41 Å, respectively, which are larger than the atomic radii of other elements (Fe: 1.24 Å, Cr: 1.25 Å, and V: 1.32 Å). When the contents of Ta and W were less than 0.4, they could be dissolved in the solid solution of BCC1 to a certain degree. Since the atomic radii of Ta and W are larger than those of other elements, the lattice distortion of the solid-solution matrix, BCC1, and the lattice constant of BCC1 increased, which inevitably appeared. Thereby, the solid-solution lattice-strain energy increased. When the content of Ta exceeds 0.4, the solid solubility of the Ta element in the solid-solution matrix of BCC1 has reached saturation. The addition of excess Ta induces the precipitation of the Laves phase. The precipitate of the second phase mitigates the lattice distortion of the solid-solution matrix to a certain extent. Hence, the lattice strain energy of the solid solution is released. An interesting phenomenon can be seen from the XRD pattern: when *x* is 1, the peak of the Laves phase is not enhanced by the previous analysis, but is slightly weakened. This feature may be related to the phase of BCC2, which is rich in W.

When *x* increased from 0.3 to 0.4, due to the precipitated Laves phase, the lattice constant of BCC2 significantly increased. When x increased to 1, the lattice constant of W drastically reduced, which may be related to the fact that W is a dominant phase, and elements, such as Fe, Cr, and V, having a small atomic radius, are dissolved in W.

### 3.2. Microstructures and Chemical Compositions

**Figure 2:**
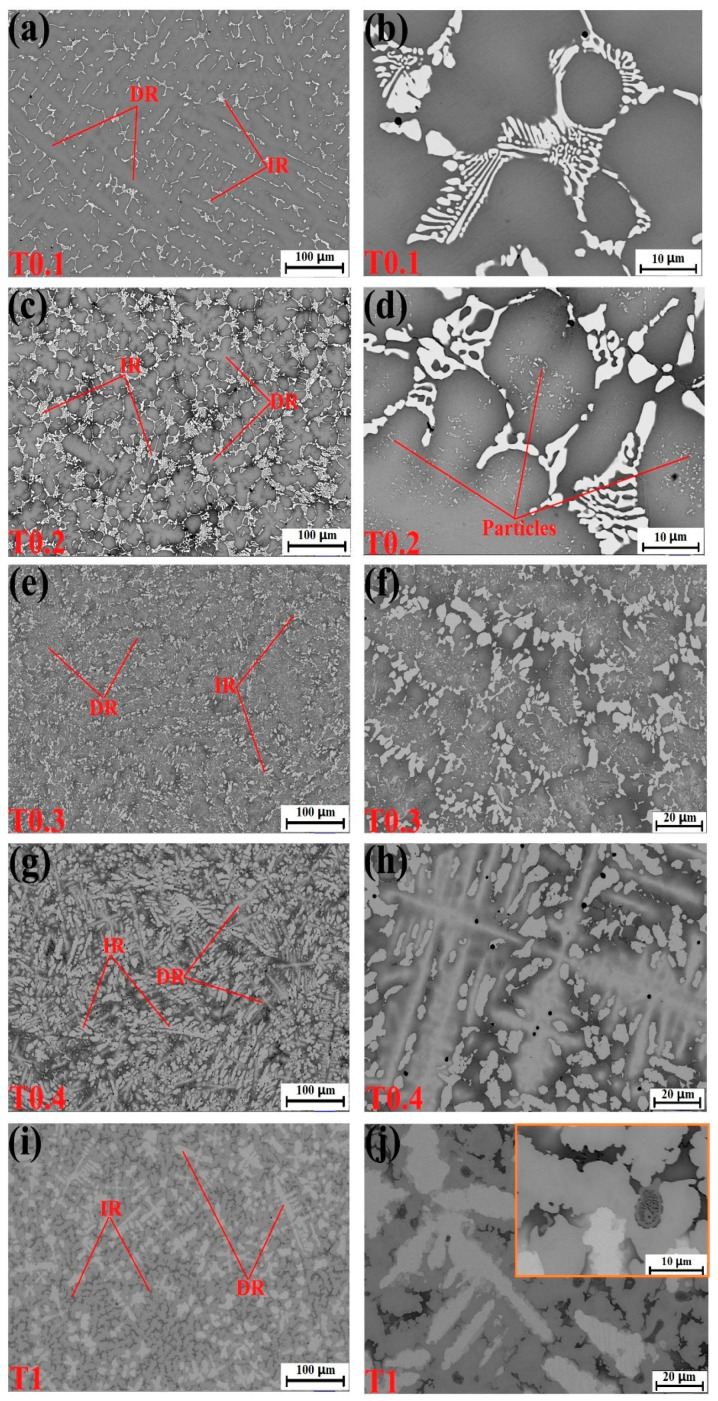
Scanning electron microscope (SEM) backscatter electron images of the as-cast alloys. (**a**,**b**) VCrFeTa_0.1_W_0.1_; (**c**,**d**) VCrFeTa_0.2_W_0.2_; (**e**,**f**) VCrFeTa_0.3_W_0.3_; (**g**,**h**) VCrFeTa_0.4_W_0.4_; and (**i**,**j**) VCrFeTaW.

The microstructures of the as-cast and as-polished VCrFeTa*_x_*W*_x_* samples captured using SEM exhibited multiple phases. Figure 2 presents the microstructures of the samples. All the alloys exhibited a typical cast dendrite (DR) structure. The EDS component analyses of the alloys are provided in Table 4. W and V were mainly present in the DR regions, Fe and Ta were concentrated in the inter-dendritic (IR) regions, whereas Cr was more evenly distributed. The distribution of elements could mainly be explained by the mixing enthalpy between elements and the difference in melting points. During the solidification of the alloys, W and V, with higher melting points, first solidified to form DR. The mixing enthalpy (Table 5) between Ta and Fe, −15 KJ/mol, which is the minimum for the selected alloy system, indicates that Ta and Fe combined together more easily than other elemental pairs in the process of solidification due to their great intermetallic compounding ability. Due to the low melting point of Fe, Fe and Ta formed in IR after the formation of DR. Ta and Fe formed a Laves phase in the solid-solution matrix composed of W and V. The microstructures of the alloy system will be described in detail later.

Figure 2a–d correspond to the microstructures of the T0.1 and T0.2 alloys. From these figures, we found that there is a small amount of the second phase in the white regions in the figures, which is the Laves phase according to the EDS results. The volume fraction of the Laves phase is lower than the XRD detection limit, as no obvious Laves phase peak was observed in the XRD pattern, as presented in Figure 1. It can be seen from the figures that they exhibit a similar DR microstructure. The IR structure of the Laves phase combined with the primary BCC1 phase in the DR region formed eutectic structures and is composed of a network-like appearance. We found that the DR size of T0.2 is less than T0.1, indicating that the addition of Ta and W can effectively reduce the size of the DR crystal grains, which is consistent with the width of the diffraction peak of T0.2, which is wider than T0.1 in the XRD pattern. The eutectic structure regions of the T0.2 alloy are significantly larger than those of the T0.1 alloy. The plate-like Laves phase thickens with increasing Ta content, meaning that the volume fraction of the Laves phase is increasing with the Ta content. This trend is consistent with the appearance of a less pronounced Laves phase around 36° on the XRD curve of T0.2 (Figure 1). From the magnified view of T0.2 in Figure 2b, there is a black area in the middle of the gray and white regions. Based on the EDS results, we found that the area is enriched by V and Fe, which was the BCC1 solid solution. On the gray DR, there are many fine precipitate particles, which is consistent with the increase in the BCC1 lattice constant.

When the content of Ta and W reached 0.3, the microstructure of the alloy still exhibited a typical DR structure (Figure 2e,f). Combined with the EDS results, the DR structure is formed by gray regions of solid-solution phases and precipitated particles of the Laves phase. The Laves phase in the IR region, with the BCC1 primary phase of the black regions in the V-rich area, formed a eutectic structure. The Laves-phase volume fraction increased significantly, which is consistent with the XRD results (Figure 1).

For the microstructure of the T0.4 alloy, shown in Figure 2g,h, the Laves phase volume fraction increased relative to T0.1, T0.2, and T0.3 alloys shown in Figure 2a–c, respectively. The Laves phases grew into a bar-like shape distributed in the matrix (Figure 2h). Combined with XRD and EDS results, the DR regions consist of a gray W-rich area of BCC2 and a black Fe–Cr–V-rich area of BCC1. The eutectic structure formed by the precipitated Laves peak of the IR structure and the primary BCC1. The distortion of the matrix was released, and the lattice constant of BCC1 reached the maximum owing to the complete precipitation of the Laves phase, which was also observed for BCC2.

The T1 alloy in Figure 2i,j had a similar appearance to T0.4, and the microstructure was a typical DR crystal structure. The white region of DR is distinctly fishbone-like shaped and is enriched in W, whereas a portion of V is a solid dissolved in the W matrix. Due to the smaller atomic radius of the V solid solution in the W matrix, the lattice constant of W decreased, which is consistent with the XRD pattern in Figure 1. The IR structure presents a hypereutectic structure, and the primary phase is not the BCC1 in the black regions, but the Laves phase in the gray regions, whereas, in the highlighted region, Laves and BCC1 phases are present in eutectic structure. These trends indicate that the additions of Ta and W not only influence the phase composition of the alloy system, but also change the microstructure of the alloy system. The addition of Ta promoted regular changes in the microstructures of the BCC1 alloy system, and the transition from the hypoeutectic structure (*x* = 0.4) to hypereutectic structures (*x* = 1) occurred.

### 3.3. Mechanical Properties

#### 3.3.1. Mechanical Properties at Room Temperature

**Figure 3:**
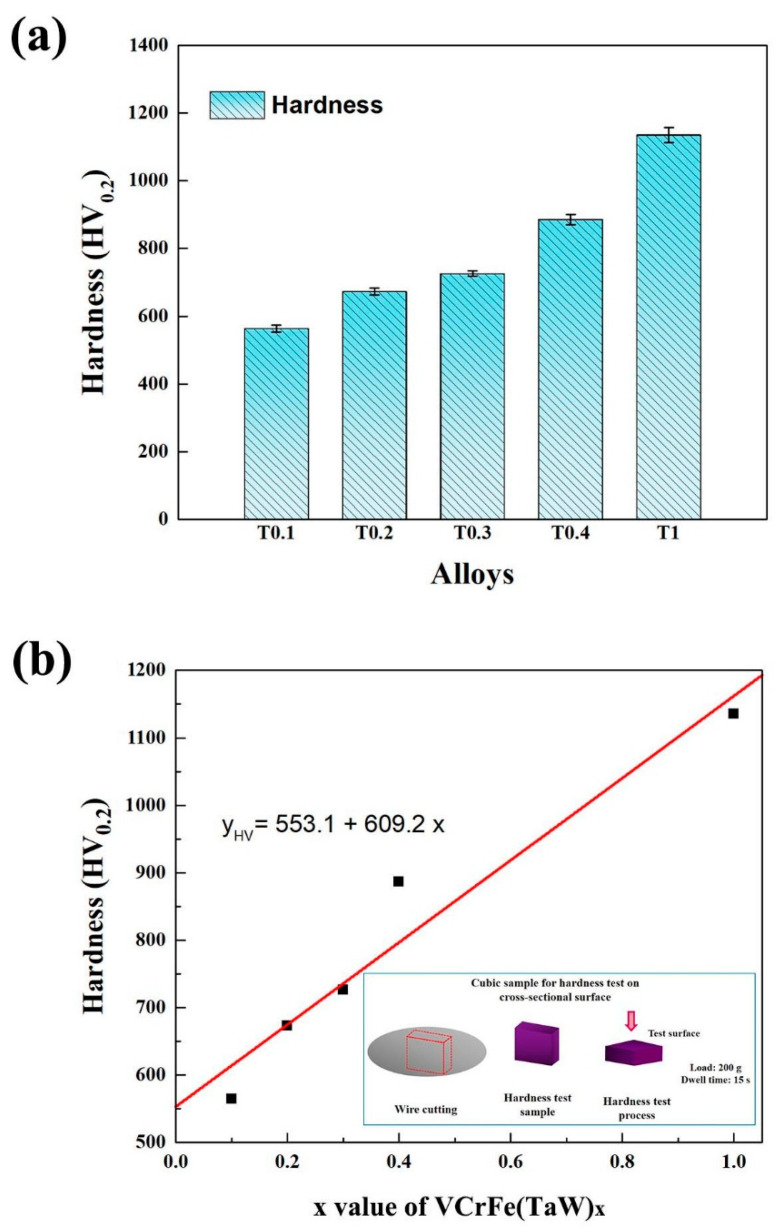
(**a**) Hardness histogram and standard deviation of the as-cast VCrFeTa*_x_*W*_x_* (*x* = 0.1, 0.2, 0.3, 0.4, and 1) alloys at room temperature; (**b**) The hardness curve of the as-cast VCrFeTa*_x_*W*_x_* (*x* = 0.1, 0.2, 0.3, 0.4, and 1) alloys as a function of the Ta and W content, and hardness test process.

Figure 3a shows a histogram of the microhardness of the alloys at room temperature and the average microhardness values are listed in Table 6. It can be seen from Figure 3a that the microhardness of the alloy system increases linearly with the increase in the Ta and W contents, from 564 HV_0.2_ of T0.1 to 1135 HV_0.2_ for the equimolar alloy, T1. The hardness enhanced with increasing *x* due to: (1) the Laves phase belongs to the intermetallic compound, and the hardness is high and (2) the addition of Ta and W caused the lattice distortion of the alloy matrix to increase continuously, and the hardness of the alloy also improved. The relationship between Ta and W content, *x*, and microhardness (y_HV_) is fitted in Figure 3b, which can be expressed as y_HV_ = 553.1 + 609.2*x*, where y is the microhardness of the alloy. The linear correlation coefficient, R, is 0.942, meaning that relationship is linear.

**Figure 4:**
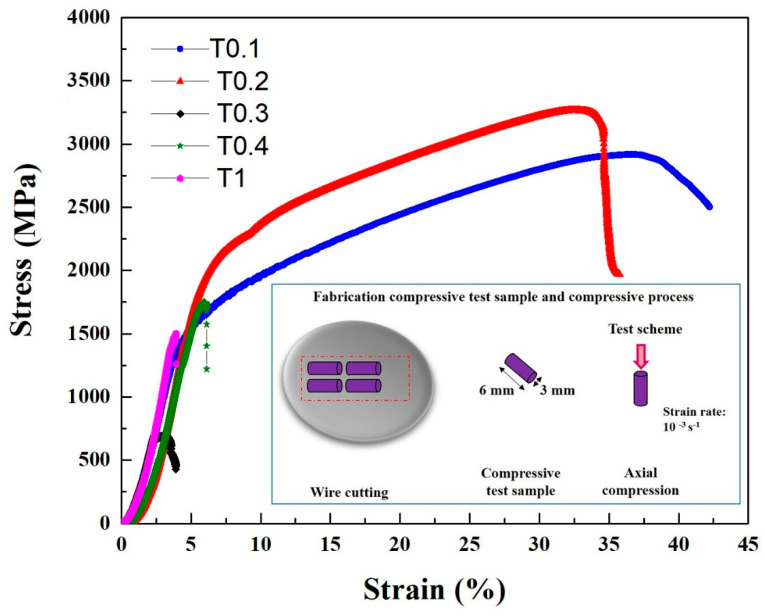
The compressive stress-strain curves of the as-cast VCrFeTa*_x_*W*_x_* (*x* = 0.1, 0.2, 0.3, 0.4, and 1) alloys at room temperature with a diameter of 3 mm.

The compressive engineering stress-strain curves are shown in Figure 4, and the compressive properties, such as yield strength σ_0.2_, fracture strength σ_bc_, and plastic strain limit ε_p_, are summarized in Table 6. Evidentially, the Ta and W content had a very pronounced effect on the compressive behavior of the alloys. It can be seen that the T0.1 and T0.2 alloys showed excellent compressive properties, with yield strength, fracture strength, and plastic strain values in T0.1 and T0.2 of 1341 MPa, 2917 MPa, and 42.2%; and 1742 MPa, 3265 MPa, and 35.7%, respectively. Compared with the compressive properties of T0.1 and T0.2, both the yield strength and fracture strength increased, which is a trade-off with plastic strain. This trend is mainly due to the presence of a large amount of dispersed fine precipitates in the T0.2 alloy and an increase in the lattice distortion, resulting in an increase in the strength of the alloy. The plastic strain decreased due to the increase in the volume fraction of the second phase. It can be seen that the compressive properties substantially decrease with the Ta and W content changing from 0.3 to 1 mainly because of the increase in the volume fraction and size of the Laves phase as the Ta content increases. The compressive performance of T0.3 is worse than T0.4 and T1, probably due to the precipitated particles growing in the matrix.

**Figure 5:**
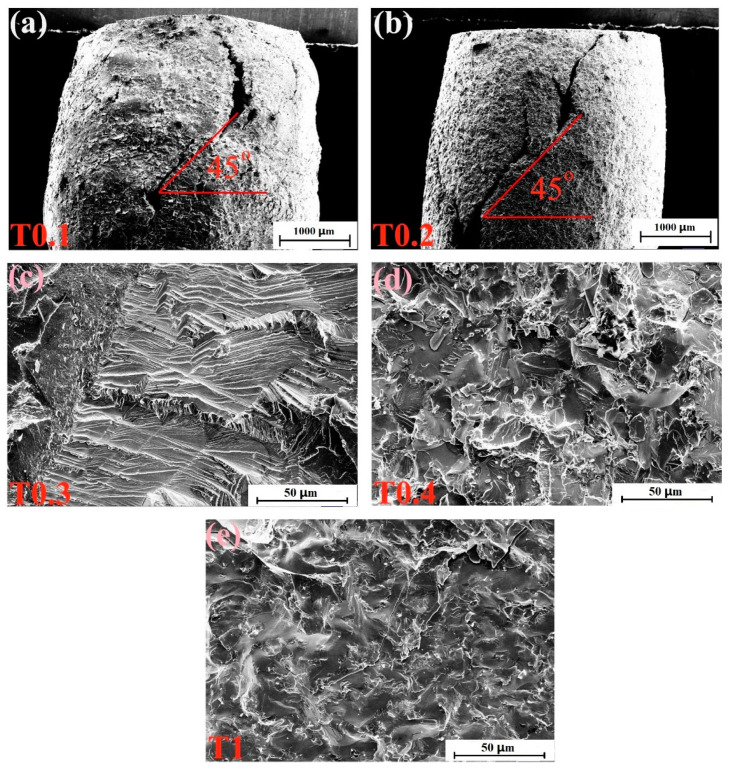
SEM micrographs of the fracture surfaces of VCrFeTa*_x_*W*_x_* (*x* = 0.1, 0.2, 0.3, 0.4, and 1) alloys at room temperature: (**a**) VCrFeTa_0.1_W_0.1_; (**b**) VCrFeTa_0.2_W_0.2_; (**c**) VCrFeTa_0.3_W_0.3_; (**d**) VCrFeTa_0.4_W_0.4_; and (**e**) VCrFeTaW.

The fracture-surface morphologies of the as-cast alloys after compressive tests at room temperature are depicted in Figure 5. Figure 5a,b show the side sections of T0.1 and T0.2 alloys, respectively. It can be seen from the figures that the alloys are not crushed, and the macroscopic appearance are waist-drum shaped. The picture of the T0.1 alloy in Figure 5a shows that the fracture surface presents severe plastic deformation, indicating its superior room-temperature plasticity and exhibiting a certain plasticity fracture. In Figure 5b, the T0.2 alloy exhibits a fracture angle of nearly 45°, and approximately parallel sliding steps of unevenness can be observed in the fracture profile. The fracture surface of the T0.3 alloy is shown in Figure 5c, which has a distinct cleavage step due to the cleavage fracture. T0.4 and T1 alloys have similar fracture patterns, and the fracture surfaces are relatively flat, demonstrating obvious tear and river patterns, which are typical quasi-cleavage fractures, in Figure 5d,e, respectively. This trend is consistent with the compressive strength of T0.4 and T1 being better than T0.3.

#### 3.3.2. Mechanical Properties at High-Temperature

**Figure 6:**
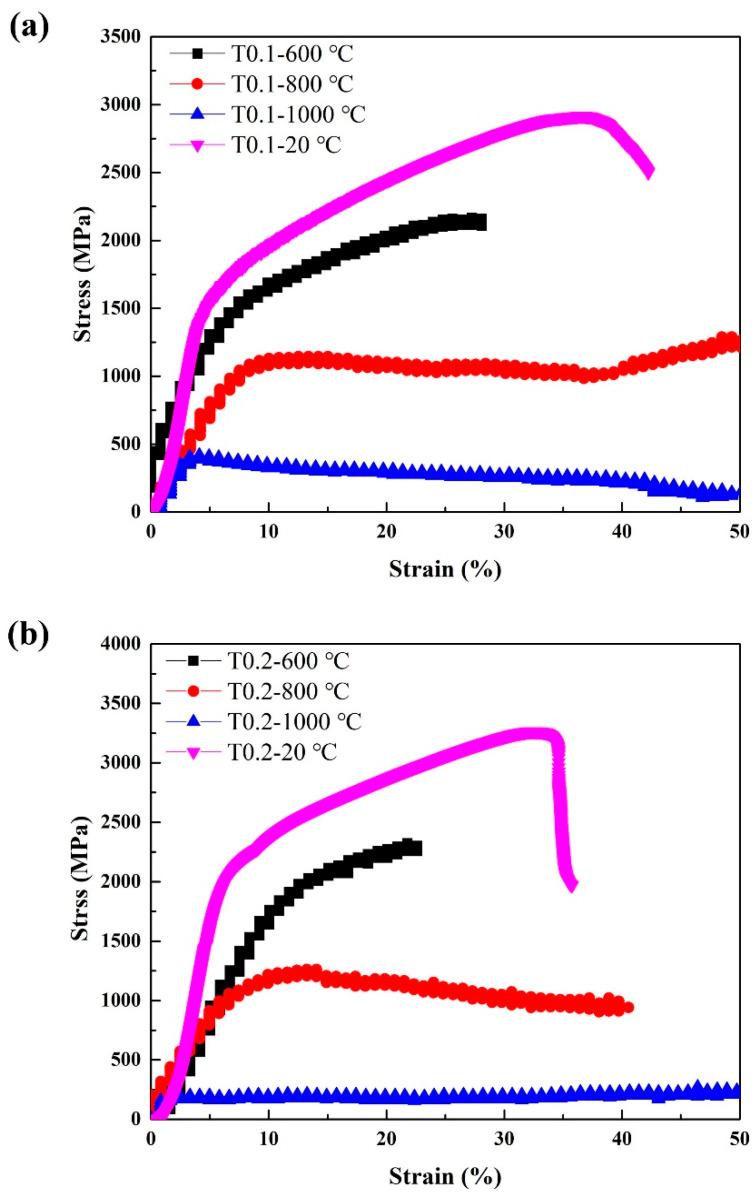
The compressive stress-strain curves of (**a**) VCrFeTa_0.1_W_0.1_ and (**b**) VCrFeTa_0.2_W_0.2_ alloys at different temperatures with a diameter of 3 mm.

Since T0.1 and T0.2 alloys exhibit excellent room-temperature strength, we also examined their high-temperature properties. Annealing at 600 °C, 800 °C, and 1000 °C for 25 h did not cause alloy softening. The hardness of the annealed T0.1 alloys are 605 HV_0.2_/ 600 °C and 621 HV_0.2_/800 °C, and T0.2 alloys are 721 HV_0.2_/600 °C and 762 HV_0.2_/800 °C, respectively, obviously higher than those of as-cast alloys presented in Table 7, indicating that the T0.1 and T0.2 alloys have the great softening resistance. Table 7 and Figure 6 show the compressive engineering stress-strain curves of T0.1 and T0.2 alloys from room temperature to 1000 °C. The strength increased and the ductility decreased with the yield strength at 600 °C. The strength decreased and the ductility increased at 800 °C. At 1000 °C, the strength of T0.1 and T0.2 alloys dropped significantly, indicating a certain degree of softening, which is also reflected in the hardness at this temperature listed in Table 7. Surprisingly, the yield strength, fracture strength, and plastic strain at 800 °C for T0.1 and T0.2 are 1019 MPa, 1289 MPa, and 50% and 1033 MPa, 1260 MPa, and 40.6%, respectively.

## 4. Discussion

### 4.1. Phase Selection

From the results, the phase structures of the VCrFeTa*_x_*W*_x_* HEAs are not as simple as presented in the XRD patterns in Figure 1. The various criteria were analyzed in order to further understand the phase formation of the alloy in this system.

Studies have shown that HEAs are prone to form solid solutions with an FCC structure or a BCC [19,21,47]. Hume-Rothery rules [21,48,49] govern the criteria for the formation of solid solutions in the binary alloy system, which include the crystal structure factor, atomic-size factor, valence-electron-concentration factor, and chemical electronegativity factor. As a special kind of the solid-solution alloy, HEAs have many components, complicating distinguishing solutes or solvents. Hence, it is difficult to study them using traditional methods [22,50]. Recent investigations extended the Hume-Rothery rules for explaining the criteria for the formation of the solid-solution structure in the HEA area with the aid of empirical relationships.

Zhang et al. [48,51] proposed three parameters affecting the formation of the HEA solid-solution phase: atomic-size difference (delta, δ), mixing enthalpy (ΔH_mix_), and mixing entropy (ΔS_mix_), to predict the phase formation in HEAs, amorphous metallic glasses, and intermetallic compounds. These calculation methods are detailed as follows:

$$\Delta H_{\mathrm{mix}}=\overset{n}{\underset{i=1,i\neq j}{\sum }}\Omega_{ij}c_{i}c_{j}$$

$$\Omega_{ij}=4{\Delta H}_{\mathrm{AB}}^{\mathrm{mix}}$$

$$\Delta S_{\mathrm{mix}}=\mathrm{klnw}=-R\overset{n}{\underset{i=1}{\sum }}(c_{i}\ln c_{j})$$

$$\delta =\sqrt{\sum_{i=1}^{n}c_{i}{\left(r_{i}-\bar{r}\right)}^{2}}$$

$$\bar{r}=\sum_{i=1}^{n}c_{i}r_{i}$$

where *n* is the number of the involved elements in an alloy, $\Delta H_{\mathrm{AB}}^{\mathrm{mix}}$ is the mixing of the enthalpy of binary equiatomic AB alloys, Ω*_ij_* is the regular melt-inter-action parameter between the *i*-th and *j*-th elements, R is the gas constant, c*_i_* and r*_i_* are the atomic percentage and atomic radius of the *i*-th element, respectively, and $\bar{r}$ is the average atomic radius. They concluded that solid solutions tend to form in the region delineated by δ > 6.6%, −15 KJ/mol ≤ ΔH_mix_ ≤ 5 KJ/mol, and 11 J/(K·mol) ≤ ΔS_mix_ ≤16.5 J/(K·mol).

To be better understand the criteria of HEAs, a new parameter Ω [21,51] was proposed to correlate the relative contribution of the change in ΔH_mix_ and ΔS_mix_, expressed as:

$$\Omega =\frac{T_{m}\Delta S_{\mathrm{mix}}}{\left|\Delta H_{\mathrm{mix}}\right|}$$

$$T_{m}=\overset{n}{\underset{i=1}{\sum }}c_{i}{\left(T_{m}\right)}_{i}$$

where (T_m_)*_i_* is the melting temperature of the *i*-th component. By analyzing the phase formation using the parameters, Ω and δ, of various reported multicomponent alloys, new parameters for forming solid-solution phases in HEAs were suggested [48]: Ω ≥ 1.1 and δ ≤ 6.6%.

HEAs often form solid solutions of an FCC or BCC phase, and the above criteria can effectively predict whether the alloy can form a solid-solution structure, but it is impossible to predict whether the solid-solution structure of the alloy is an FCC or BCC phase. Guo et al. [52] proposed the relationship between the valence-electron concentration (VEC) and solid-solution stability. The VEC in a multi-component system is:

$$\mathrm{VEC}=\overset{n}{\underset{i=1}{\sum }}c_{i}{\left(\mathrm{VEC}\right)}_{i}$$

where (VEC)*_i_* is the VEC of the *i*-th element. From published experimental results [37,52], the limitation that they suggested were: FCC phases occur at VEC ≥ 8.0, BCC phases at VEC < 6.87, and a mixture of FCC and BCC phases at 6.87 ≤ VEC < 8.

**Figure 7:**
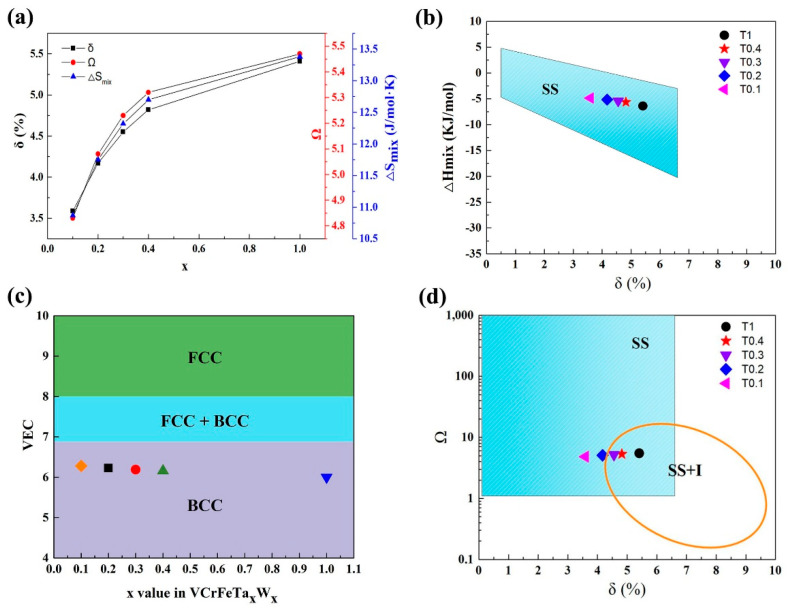
(**a**) The curves of δ, Ω, and ΔS_mix_ as a function of the Ta and W content for VCrFeTa*_x_*W*_x_* (*x* = 0.1, 0.2, 0.3, 0.4, and 1) alloys; (**b**) The relationship between parameters δ and ΔH_mix_ for the as-cast VCrFeTa*_x_*W*_x_*; (**c**) The relationship between the valence-electron concentration (VEC), and Ta and W content of VCrFeTa*_x_*W*_x_* alloys; (**d**) The relationship between the parameters δ and Ω, for the as-cast VCrFeTa*_x_*W*_x_*. (SS: Solid-Solutions; I: Intermetallics compound; SS + I: Solid-Solutions + Intermetallics compound).

For the VCrFeTa*_x_*W*_x_* alloys in the present work, we studied the phase-formation rules according to the above parameters. The specific results are shown in Table 8. It can be seen from the table that as the contents of Ta and W increase, the δ of the alloy system grows from 3.59% to 5.41%, as plotted in Figure 7a. This indicates that the degree of the lattice distortion caused by the atomic arrangement is increasing, which is consistent with the lattice constants of the two phases when x is between 0.1 and 0.4 (the lattice constant decreases in the case of the equimolar HEA, which is related to the Laves phase being the primary phase, and V is distributed into a solid-solution with W). Despite the change in δ and ΔH_mix_ with the increase in the Ta and W content, their values meet the requirement for forming solid solutions, as plotted in Figure 7a,b, indicating that the mixing entropy effect of the alloy is stronger than that of the mixing enthalpy. The tendency of the alloy to form solid-solutions is improved, indicating that the effect of the mixing of entropy on the solid solution formation strengthens. The VEC in the studied alloys is shown in Figure 7c. With increasing Ta and W addition, the value of VEC decreased from 6.28 to 6, which meets the BCC-forming requirement (6.87 ≤ VEC < 8), demonstrating that the BCC phase is stable in the VCrFeTa*_x_*W*_x_* alloy system.

With the increase in the Ta and W content, although both ΔS_mix_ and Ω (≥ 1.1) increased (Figure 7a), from Figure 7d, T0.3, T0.4, and T1 HEAs are in the SS + I (SS: Solid-solution, I: Intermetallics compound) region. The mixing of the enthalpy promotes the formation of intermetallic compounds, whereas the Ta–Fe binary system has the most negative mixing of enthalpy (−15 kJ/mol) listed in Table 5, indicating that the bonding force between these two elements is the strongest. The Fe_2_Ta phase formation occurs, which is consistent with the XRD patterns in Figure 1.

### 4.2. Ta and W Effects at Room Temperature

The microstructure analysis of the VCrFeTa*_x_*W*_x_* alloys with different Ta and W contents, performed in the current research, revealed several features. First, an increase in the Ta and W content substantially decreased the BCC1 matrix phase of the alloys (Figure 1). Conversely, the volume fraction of BCC2 and Laves phases increase. Second, an addition of Ta and W resulted in the formation of intermetallic phases, namely the Laves phase of Fe_2_Ta-type. The Laves phase can be associated with a highly negative-enthalpy of the intermetallic-phases formation [19,37] (Table 6). Third, with increasing Ta and W contents, the change of precipitation was the most obvious and most significant phenomenon. When *x* = 0.1, the Laves precipitation phase has a lamellate shape and forms an eutectic structure with the matrix. When *x* = 0.2, the Laves-precipitation phase is thicker, and still forms an eutectic structure with the matrix, and fine particles, most of which are the Laves phase precipitated on the matrix. When *x* = 0.3–1, the precipitation phase gradually aggregates and grows. These results indicate that the addition of Ta and W not only changes the phase composition of the alloy system, but also varies the microstructure of the alloy system. However, the addition of Ta promoted the regular Laves phase microstructure change of the BCC1 alloy system, and a transition from the hypoeutectic structure (*x* ≤ 0.4) to hypereutectic structure (*x* = 1) occurred. This indicates that Ta can promote the eutectic transformation of the Fe–Cr–V alloy system.

The Ta and W contents have a prominent effect on the mechanical properties of the VCrFeTa*_x_*W*_x_* alloys, as expected given the pronounced changes in the microstructure. With the addition of Ta and W, the hardness of the alloy increases linearly, while the plasticity and strength of the studied alloys exhibited a complex dependence. In the HEA solid-solution phase, various atoms randomly occupy the lattice position of the crystal. Each atom is surrounded by other kinds of atoms, and all atoms can be regarded as solute atoms or solvent atoms [37,53]. In addition, the types of atoms vary in size, causing severe lattice distortion in the solid solution, which in turn leads to high solid-solution strengthening [46,54,55], thereby increasing the strength and hardness of the alloy, especially the HEAs of the BCC structure [24,56,57,58]. Conversely, W obviously promotes the formation of BCC2, and Ta prefers forming Laves phases. The mixing enthalpy between Ta and Fe is −15 kJ/mol (Table 5), which is the lowest for the VCrFeTa*_x_*W*_x_* system, meaning that Ta and Fe combine together quite easily compared with the other element pairs during solidification owing to their great compatibility. The hardness values of the VCrFeTa*_x_*W*_x_* alloys increase with increasing *x*. As the content of Ta and W increased, the yield strength increased from T0.1 to T0.2. However, in the VCrFeTa*_x_*W*_x_* (*x* = 0.3, 0.4, and 1) alloys, the yield strength deteriorated quickly with an increase in *x* (Figure 4). This phenomenon can be associated with a large volume fraction of Laves phases, and the size of Laves particles increases with the change in *x*. Notably, the strengthening contribution of the precipitation particles to the BCC phase alloys was prominent, which has been previously studied [59]. In the present study, the fine second phase particles of the Laves phase even had a positive effect on the strength, as the T0.2 alloy has an excellent yield strength and plasticity.

**Figure 8:**
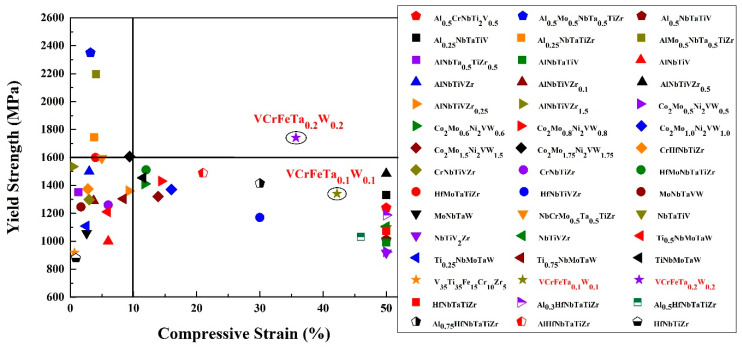
The map of compressive yield strength and ductility combinations of various refractory high-entropy alloys [13,24,29,56,57,58,59,60,61,62,63,64,65,66,67,68,69,70,71,72] at room temperature. Initial strain rates range from 10^−4^ to 10^−3^ s^−1^.

As engineering materials, the yield strength of HEAs is an important parameter for the design of a component. The dependence of the room-temperature compressive yield strength of the reported HEAs [13,24,29,56,57,58,59,60,61,62,63,64,65,66,67,68,69,70,71,72] and the studied alloys in the present work are plotted in Figure 8. The T0.1 and T0.2 HEAs possess relatively-high yield strength, whereas they all exhibit excellent compressive strains. T0.1 and T0.2 have a yield strength of 1341 MPa and 1742 MPa with plastic strains of 42.2% and 35.7%, respectively. They exhibit excellent plasticity, as they could not be broken in the compressive tests. The refractory HEAs reported in papers exhibit high yield strength, while their plastic strains are lower than 15%; or the reported refractory HEAs show remarkable plastic strains more than 50%, while their yield strengths are not remarkable. If we set the coordinates of the alloys in Figure 8 at (10%, 1600 MPa), only the T0.2 alloy is in the first quadrant. Therefore, we concluded that the BCC HEAs developed so far still have insufficient compressive strain at room temperature, or exhibit low strength. It is noteworthy that T0.2 studied in the current work not only possesses a compressive strain up to 35.7%, but also has a yield strength as high as 1742 MPa. In other words, T0.2 may have excellent ductility, which makes this kind of HEA better than other HEAs for engineering applications. More significantly, the T0.2 alloy, as a low-activation HEA, has obvious performance advantages over the previously-reported low-activation alloys, indicating that it has potential as a candidate material for fusion reactors.

### 4.3. Heat-Softening Resistance

**Figure 9:**
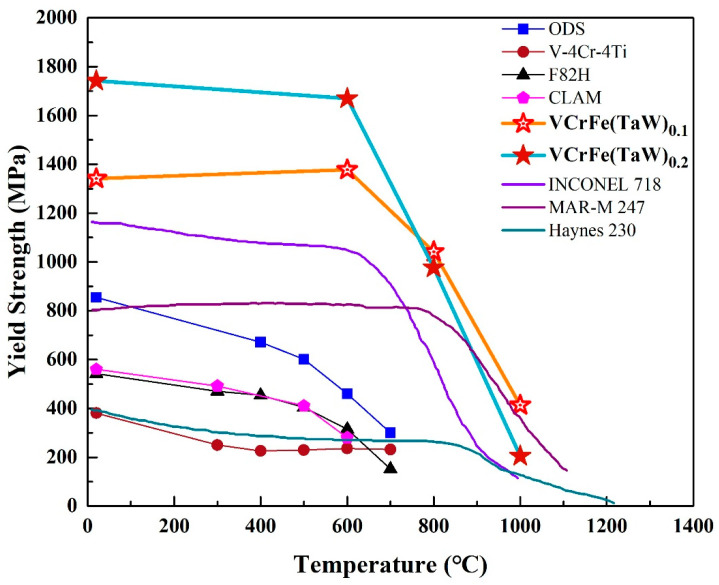
Temperature dependence of the compressive yield strength of superalloys [37], reported HEAs [36,68], and low activation alloys [13].

Typically, annealing leads to alloy softening due to the rapid diffusion of atoms, resulting in internal stress relaxation caused by alloy defects at high temperatures [28,29]. However, due to the high mixing entropy and the obvious sluggish diffusion effect, HEAs have good high-temperature stability and great resistance to high temperature softening [28,35,37]. According to the Gibbs free energy formula, with the increase in temperature, the effect of entropy is more obvious than that of enthalpy [21]. The high mixing-entropy increases the solid solubility of the alloy, which is conducive to the formation of the solid-solution structure. Because of the sluggish diffusion effect, the alloy easily forms a supersaturated solid solution and a fine precipitated phase. Annealing at elevated temperatures can partially release the internal stress and dissolve atoms. For the studied alloys in the present work, the increase in hardness after annealing from 600 °C to 800 °C is due to solid-solution strengthening. Although the microhardness of the T0.1 and T0.2 alloys decreased at 1000 °C, the reduction was not large compared to the hardness values at room temperature. For traditional alloys, tempering after quenching tends to result in significant softening. However, HEAs have significant advantages in this regard. HEAs, such as NbMoTaW [56], NbMoTaWV [56], and AlNbTiV [68], with a typical BCC structure have been reported to show that, although the alloys have poor compressive strains at room temperature, the plasticity of the alloys increases as the temperature rises. As shown in Figure 9, from room temperature to 800 °C, the yield strength of the T0.1 and T0.2 alloys are significantly higher than those of superalloys, such as Inconel 718 and Haynes 230. Although the strength of MoNbHfZrTi is higher than those of the T0.1 and T0.2 alloys at room temperature, its compression plasticity is only 10.1%, far lower than the T0.1 and T0.2 alloys. However, the strengths of T0.1 and T0.2 alloys exceed the reported high-entropy alloys include MoNbHfZrTi at 600–800 °C [36,68]. Thereby, the HEAs exhibit excellent resistance to high-temperature softening. As an alternative alloy for future fusion reactors with the low activation, the target alloys in the present work have certain advantages at high temperatures compared to CLAM, ODS, and V–4Cr–4Ti, as presented in Figure 9.

## 5. Summary

Novel low-activation HEAs VCrFeTa*_x_*W*_x_* (*x* = 0.1, 0.2, 0.3, 0.4, and 1) were fabricated by vacuum arc melting, and their microstructure and mechanical properties were studied in the present work. The investigated alloys exhibited a relatively simple microstructure and promising properties. Based on the obtained results and discussions, our conclusions are as follows:

(1) The microstructures of all investigated alloys exhibited a typical dendritic and eutectic structure, with VCrFeTa_0.1_W_0.1_ and VCrFeTa_0.2_W_0.2_ presenting a mainly BCC1 (a VCrFe-rich region) solid solution and Laves phases (an Fe_2_Ta-type). VCrFeTa_0.3_W_0.3_, VCrFeTa_0.4_W_0.4_, and VCrFeTaW contain BCC1 and BCC2 (W-rich region) solid solutions and Laves phases.

(2) The Vickers hardness of the alloys increased with increasing Ta and W contents. The hardness values of VCrFeTa_0.1_W_0.1_, VCrFeTa_0.2_W_0.2_, VCrFeTa_0.3_W_0.3_, VCrFeTa_0.4_W_0.4_, and VCrFeTaW are 546 HV_0.2_, 673 HV_0.2_, 726 HV_0.2_, 886 HV_0.2_, and 1135 HV_0.2_, respectively. This feature is attributed to the solid-solution strengthening and the increased amount of Laves and BCC2 phases.

(3) The augmented Ta and W contents increased the compressive strength but decreased the plastic strain of T0.1 and T0.2 alloys. The T0.1 and T0.2 alloys exhibited compressive yield strengths of 1341 MPa and 1742 MPa, with plastic strains of 42.2% and 35.7%, respectively. The solid-solution strengthening of the BCC matrix and the formation of hard Laves phases precipitated in particles are two main factors contributing to alloy strengthening. With the addition of Ta and W, the compressive performance deteriorated sharply due to the increase in the volume fraction and the growth of the Laves phase.

(4) By applying the atomic ratios strategy and the criteria for disordered solid solutions, the alloy system of VCrFeTa_0.1_W_0.1_, VCrFeTa_0.2_W_0.2_, VCrFeTa_0.3_W_0.3_, VCrFeTa_0.4_W_0.4_, and VCrFeTaW fully met the VEC, δ-Ω, and δ-△H_mix_ criteria. Studies have shown that HEAs maintain stable phase structures and properties after high-temperature annealing. This means that the design of high-temperature structural materials using the HEA concept could promote the development of these alloys for use in extreme high-temperature extreme such as blades, engines, aerospace, fusion reactors and other applications.

(5) The high-temperature mechanical properties of T0.1 and T0.2 alloys were examined. After annealing 25 h at 600–1000 °C, the T0.1 and T0.2 alloys maintained high hardness. The compressive yield strengths of the T0.1 and T0.2 alloys are promising with heat-softening resistance at 600–800 °C. The yield strengths of T0.1 and T0.2 alloys were much higher than 1000 MPa at 800 °C. This study provides guidance for further development of the current concepts to produce refractory high-temperature structural materials, which are candidate alloys for fusion reactors.

## Figures and Tables

**Table 1:** Low activation elements and high activation elements.

**Low activation element**	Fe	Ti	Cr	V	Ta	Zr	W	Mn	Si	Al	B	C	N	O
**High activation element**	Nb	Ni	Co	Cu	Mo									

**Table 2:** Nominal compositions of experimental alloys (Nominal compositions, at. %).

Alloy	Identification	Fe	Cr	V	Ta	W
VCrFeTa_0.1_W_0.1_	T0.1	31.2	31.2	31.2	3.2	3.2
VCrFeTa_0.2_W_0.2_	T0.2	29.4	29.3	29.3	6	6
VCrFeTa_0.3_W_0.3_	T0.3	27.8	27.7	27.7	8.4	8.4
VCrFeTa_0.4_W_0.4_	T0.4	26	26	26	11	11
VCrFeTaW	T1	20	20	20	20	20

**Table 3:** Phase compositions of the VCrFeTa*x*W*x* alloy system and lattice constants of the solid-solution phase.

Alloy	Phase Composition	Lattice Constant (nm)
VCrFeTa_0.1_W_0.1_	BCC1	0.2935
VCrFeTa_0.2_W_0.2_	BCC1	0.2937
VCrFeTa_0.3_W_0.3_	BCC1	0.2947
	BCC2	0.3174
VCrFeTa_0.4_W_0.4_	BCC1	0.2963
	BCC2	0.3178
	Laves	-
VCrFeTaW	BCC1	0.2962
	BCC2	0.3166
	Laves	-

**Table 4:** Chemical compositions in different regions of various alloys by energy-dispersive spectroscopy (EDS) (at. %).

Alloy	Region		V	Cr	Fe	Ta	W
VCrFeTa_0.1_W_0.1_		Overall	33.29 ± 0.29	30.94 ± 0.17	30.75 ± 0.22	2.54 ± 0.27	2.47 ± 0.02
	IR	White	19.66 ± 0.30	22.26 ± 0.35	37.57 ± 0.52	20.51 ± 0.79	/
	DR	Gray	35.98 ± 0.30	33.32 ± 0.35	24.80 ± 0.52	2.20 ± 0.24	3.70 ± 0.40
VCrFeTa_0.2_W_0.2_		Overall	31.15 ± 0.21	29.97 ± 0.31	28.58 ± 0.40	5.01 ± 0.59	5.29 ± 0.08
	IR	White	15.98 ± 0.18	23.30 ± 0.21	38.75 ± 0.32	21.96 ± 0.49	/
	DR	Gray	35.57 ± 0.21	33.72 ± 0.19	21.95 ± 0.21	2.76 ± 0.31	6 ± 0.07
	DR	Black	32.41 ± 0.18	26.72 ± 0.16	39.49 ± 0.24	1.39 ± 0.11	/
VCrFeTa_0.3_W_0.3_		Overall	30.27 ± 0.20	28.54 ± 0.19	28.52 ± 0.52	6.21 ± 0.37	6.36 ± 0.04
	IR	White	10.33 ± 0.25	22.54 ± 0.62	42.89 ± 0.61	24.24 ± 0.14	/
	DR	Gray	24.75 ± 0.28	31.51 ± 0.59	31.36 ± 0.60	/	12.38 ± 0.10
	DR	Black	44.96 ± 0.36	23.82 ± 0.54	25.74 ± 0.65	5.48 ± 0.07	/
VCrFeTa_0.4_W_0.4_		Overall	27.54 ± 0.20	27.57 ± 0.21	27.19 ± 0.26	9.09 ± 0.43	8.62 ± 0.07
	IR	White	15.19 ± 0.28	23.59 ± 0.66	36.01 ± 0.63	25.20 ± 0.15	/
	DR	Gray	28.49 ± 0.54	26.94 ± 0.66	25.51 ± 0.62	/	19.06 ± 0.13
	DR	Black	28.88 ± 0.28	28.43 ± 0.52	36.17 ± 0.60	/	6.52 ± 0.07
VCrFeTaW		Overall	22.12 ± 0.34	18.99 ± 0.71	20.99 ± 0.65	19.00 ± 0.82	18.90 ± 0.76
	DR	White	13.08 ± 0.50	7.76 ± 0.59	/	/	79.16 ± 0.39
	IR	Gray	14.64 ± 0.30	22.23 ± 0.41	30.96 ± 0.63	32.22 ± 0.18	/
	IR	Black	39.21 ± 0.60	25.32 ± 0.69	35.48 ± 0.76	/	/

**Table 5:** The formation enthalpies between elements (KJ/mol).

	Fe	Cr	V	Ta	W
Fe	-	−1	−7	−15	0
Cr	-	-	−2	−7	1
V	-	-	-	−1	−1
Ta	-	-	-	-	−7
W	-	-	-	-	-

**Table 6:** Mechanical properties of the as-cast VCrFeTa*x*W*x* (*x* = 0.1, 0.2, 0.3, 0.4, and 1) alloys.

Alloy	Vickers Hardness (HV_0.2_)	$\sigma_{0.2}\;(\mathrm{MPa})$	$\sigma_{bc}\;(\mathrm{MPa})$	$\varepsilon_{p}\;(\%)$
VCrFeTa_0.1_W_0.1_	564	1341	2917	42.2 (Not broken)
VCrFeTa_0.2_W_0.2_	673	1742	3265	35.7 (Not broken)
VCrFeTa_0.3_W_0.3_	726	/	701	/
VCrFeTa_0.4_W_0.4_	886	1580	1767	/
VCrFeTaW	1135	/	1501	/

σ_0.2_: yield strength; $\sigma_{\mathrm{bc}}$: ultimate compressive strength; and ε_p_: plastic-strain limit.

**Table 7:** Mechanical properties of T0.1 and T0.2 alloys at 600–1000 °C.

Alloy	Temperature (°C)	σ_0.2_(MPa)	$\sigma_{bc}$ MPa	ε_p_ (%)	Vickers Hardness (HV_0.2_)
VCrFeTa_0.1_W_0.1_	600	1234	2158	28.1	605
	800	1019	1289	> 50	621
	1000	371	421	> 50	538
VCrFeTa_0.2_W_0.2_	600	1657	2316	22.6	721
	800	1033	1260	40.6	762
	1000	182	253	> 50	665

Note: σ_0.2_: yield strength, $\sigma_{\mathrm{bc}}$: ultimate compressive strength, and ε_p_: plastic-strain limit.

**Table 8:** ΔH_mix_, ΔS_mix_, Ω, δ, valence-electron concentration (VEC), the theoretical density, and the melting points for VCrFeTa*_x_*W*_x_* (*x* = 0.1, 0.2, 0.3, 0.4, and 1) alloys.

Alloy	δ	ΔH_mix_ (KJ/mol)	ΔS_mix_ (J/mol·K)	Ω	VEC	ρ_theor_ (g/cm^3^)	T_m_ (K)
VCrFeTa_0.1_W_0.1_	3.59	–4.83	10.87	4.83	6.28	7.85	2147.7
VCrFeTa_0.2_W_0.2_	4.17	–5.15	11.75	5.08	6.23	8.58	2227.3
VCrFeTa_0.3_W_0.3_	4.55	–5.41	12.32	5.23	6.19	9.19	2297.0
VCrFeTa_0.4_W_0.4_	4.82	–5.63	12.70	5.32	6.16	9.72	2360.1
VCrFeTaW	5.41	–6.4	13.38	5.47	6	11.81	2631.8
